# Three-times-weekly, post-dialysis cefepime therapy in patients on maintenance hemodialysis: a retrospective study

**DOI:** 10.1186/s40360-016-0048-y

**Published:** 2016-02-04

**Authors:** Eric Descombes, Filipe Martins, Ould Maouloud Hemett, Veronique Erard, Christian Chuard

**Affiliations:** Service of Nephrology, Department of Internal Medicine, HFR Fribourg-Hôpital Cantonal, Fribourg, Switzerland; Service of Infectious Diseases, Department of Internal Medicine, HFR Fribourg-Hôpital Cantonal, Fribourg, Switzerland

**Keywords:** Hemodialysis, Beta-lactam antibiotics, Cefepime, Pharmacokinetics

## Abstract

**Background:**

In hemodialysis patients, post-dialysis treatment with intravenous antibiotics permits even severe infections to be managed on an outpatient basis. Cefepime is a fourth-generation cephalosporin with a broad spectrum of action in monotherapy. We report on the pharmacokinetics of cefepime in post-dialysis therapy.

**Methods:**

Since June 2012, twelve infections were treated with post-dialysis cefepime in 9 patients on high-flux hemodialysis. The initial post-dialysis dose of cefepime was approximately 15 mg/kg. The following doses were adapted according to the trough serum levels obtained before the subsequent dialysis in order to be above the EUCAST breakpoints for susceptible organisms and above the MIC90. Residual plasma concentrations were determined before (*n* = 30) and after (*n* = 17) dialysis by liquid chromatography–mass spectrometry.

**Results:**

Overall, the mean ± SD dose of cefepime was 920 ± 270 mg (14.5 ± 5.1 mg/kg), but it was significantly lower before the 48 h interval (775 ± 210 mg or 12.7 ± 4.5 mg/kg) compared to the 72 h interval (1125 ± 225 mg or 17.2 ± 4.9 mg/kg) (*p* < 0.05). The mean trough pre-dialysis concentrations were 10.7 ± 3.9 mg/l and 11.3 ± 5.6 mg/l at 48 and 72 h, respectively. These levels always largely exceeded the EUCAST susceptibility breakpoints for all the targeted bacteria (>1 mg/l) with the exception of Pseudomonas aeruginosa (>8 mg/l). Cefepime concentrations were higher in anuric patients compared to those with preserved diuresis (15.6 ± 3.5 vs 9.25 ± 3.6 mg/l; *p* < 0.001) and decreased on average by 81 % during dialysis (from 10.5 ± 3.7 to 1.96 ± 1.2 mg/l; *p* < 0.001). The clinical outcome of all patients was good.

**Conclusions:**

Outpatient treatment with cefepime administered post-dialysis three-times-weekly was effective and well-tolerated in our patients. According to our data, in patients infected by highly susceptible pathogens a fixed dose of cefepime of 1 g before every 48-h interval and of 1.5 g before every 72-h interval should be recommended, without need of routine monitoring of the cefepime blood levels. In patients having an infection with less susceptibles pathogens as P. aeruginosa, and particularly in those among them exhibiting residual renal function, higher initial doses are necessary (1.5 g before a 48-h interval and 2.0 g before a 72-h interval) with adaption according to the subsequent pre-dialysis trough serum levels.

## Background

Chronic hemodialysis (HD) patients are at a high risk of developing infectious complications due to their fragility and state of relative immunodeficiency [1–3]. The need for vascular access (i.e. autologous fistula, prosthetic graft or central venous catheter) also increases this risk and infection remains the second leading cause of death after cardiovascular disease in HD patients [1–5]. Managing severe infections most often requires intravenous (IV) antibiotics and thus these patients must be hospitalized for IV treatment. The use of post-dialysis therapy is interesting, because it facilitates the option of managing even severe infections with IV antibiotics on an outpatient basis. This may shorten or avoid hospitalization and improve the quality of life while in the interim reducing treatment costs. Furthermore, prescribing an IV antibiotic after dialysis is associated with 100 % compliance which is clearly better than the estimated compliance of approximately 70 % associated with taking an oral medication [6–8].

Because of its very prolonged half-life in HD patients, post-dialysis vancomycin has been used in dialysis centers for a long time [9]. However, vancomycin has a narrow spectrum of action which is limited to Gram-positive microorganisms. Therefore, some years ago, we also started to use post-dialysis IV ceftriaxone with quite satisfactory results [10]. Since reimbursement for ceftriaxone in outpatient therapy is restricted in Switzerland, we subsequently considered the possibility of using cefepime instead. Cefepime is a fourth-generation cephalosporin developed in the early 1990s with a broad spectrum of action against both Gram-positive and Gram-negative bacteria including Enterobacter sp., Citrobacter sp. and Pseudomonas aeruginosa, but without an effect on strictly anaerobic bacteria or resistant strains such as methicillin-resistant Staphylococcus aureus (MRSA) and extended spectrum beta-lactamase-producing (ESBL) Enterobacteriaceae [11, 12]. Like the other beta-lactams, it acts by linking the penicillin-binding-protein and inhibiting synthesis of the peptidoglycan which leads to defects in the bacterial wall and cell death. The pharmacodynamics of beta-lactam antibiotics are based on time-dependent bactericidal activity [13].

Cefepime is primarily eliminated by the kidney in unchanged form (85 %) with a half-life of approximately 2 h in subjects with normal renal function, but elimination is much longer - up to 22 h - in anuric patients on dialysis [14]. Cefepime hydrochloride has a molecular weight of 571, is only slightly bound to plasma proteins (16-19 %) and is quite effectively cleared by dialysis [14, 15]. Concerning its use in HD patients, recommendations in the literature differ greatly, but in general the daily administration of a single reduced dose is recommended. Cronquist et al. propose administering 250 mg of cefepime daily [14], Bennett et al. recommend that the standard daily dose be reduced by 50–75 % [16] and official Swiss recommendations advise a dose of 500 mg once daily [17].

Pharmacodynamic studies of cefepime in patients with impaired renal function in the early 1990s already suggested that this antibiotic could be prescribed every 48 h in dialyzed patients [14]. However, there are only 2 reports concerning the post-dialysis use of cefepime and these are both from the same Austrian group [18, 19]. They propose prescribing a fixed post-dialysis dose of 2 g three-times-weekly, but it should be noted that this dosage was associated with relatively high mean residual cefepime serum concentrations, e.g. 23 ± 7 mg/l [18], that may be associated to an increased risk of neurotoxicity [20–22].

In light of all these data, we decided in 2012 to use post-dialysis IV cefepime when indicated and to adapt the prescribed dose according to the pre-dialysis trough serum levels, adopting a therapeutic drug monitoring (TDM) approach. The objective of the present study is to report a retrospective analysis of our experience as well as the pharmacokinetics of cefepime after post-dialysis administration three times each week.

## Methods

From June 2012 to December 2013, twelve cases of infection which occurred in 9 HD patients of our dialysis unit were treated with post-dialysis cefepime. In all these patients cefepime was considered as a first choice antibiotic based on the clinical and microbiological data and was prescribed at the appropriate dose (based on the TDM approach used) as part of our standard clinical cares. The medical records of these nine patients were studied retrospectively, and all subjects retrospectively gave their informed consent authorizing us to use their demographic and medical data. According to the rules of our Hospital based on the Cantonal Law (Loi du 16 novembre 1999 sur la santé; http://bdlf.fr.ch/frontend/versions/4139?locale=fr 4139?locale = fr) the present study, being retrospective, did not require formal review by the Ethics Committee of our Institution (Commission d'éthique pour les projets de recherche biomédicale du Canton de Fribourg).

These nine patients (4 males/5 females) were dialyzed due to chronic kidney disease associated with diabetic (*n* = 4) and vascular (*n* = 1) nephropathy, glomerulonephritis (*n* = 1), amyloidosis (*n* = 1) and multifactorial CKD (*n* = 2). Their mean age and weight were 68.9 ± 6.8 years and 62.0 ± 11.9 kg, respectively (mean BMI 23.0 ± 3.5). Four of these patients were anuric and the rest had a residual diuresis >400 ml/day (mean diuresis: 950 ± 250 ml/24 h; mean residual creatinine clearance: 3.2 ± 2.4 ml/min). All were dialyzed three times each week with high-flux HD using one of the following high-flux dialyzers: Polyflux 170H (Gambro), FX80 (Fresenius) or Sureflux 190UX (Nipro). Dialysis sessions were on average 4 h long, with blood flow rates averaging 300 ml/min and a dialysate flow of 500 ml/min.

The sites of the treated infections were: lungs (*n* = 4), urinary tract (*n* = 3), catheter-related (*n* = 2), skin, bone and digestive tract (one each). The causal pathogens were identified in 7 cases in sputum (*n* = 2), wound smear (*n* = 1), abscess (*n* = 1), urine (*n* = 2) and blood (*n* = 1).

The initial post-dialysis dose of cefepime (Cefepime OrPha®, Orpha Swiss GMBH, Switzerland) was approximately 15 mg/kg and ranged from 750 to 1500 mg (given as a 5–10 min infusion at the end of dialysis). The higher doses of 1500 mg were mainly prescribed before the 72-h interval of a weekend. The subsequent doses were adapted when appropriate according to the trough serum levels obtained before the following HD session in order to remain above the European Committee on Antimicrobial Susceptibility Testing (EUCAST) breakpoints for susceptible organisms [23, 24] and above the MIC90 of a clinical collection of bacteria [11].

The residual plasma concentrations of cefepime were determined before (*n* = 30) and after (*n* = 17) HD. The cefepime concentrations were measured by liquid chromatography-mass spectrometry (LC-MS/MS) in the Clinical Pharmacology Laboratory, CHUV University Hospital, Lausanne, Switzerland. The cefepime trough levels generally recommended by this laboratory range from 2 to 15 mg/l.

### Statistical methods

The results are given as mean ± standard deviation. For continuous variables, the difference between two groups was assessed by the student’s t-test for unpaired data. All tests were two-sided and significance was deemed to exist for *p* < 0.05.

## Results

**Fig. 1:**
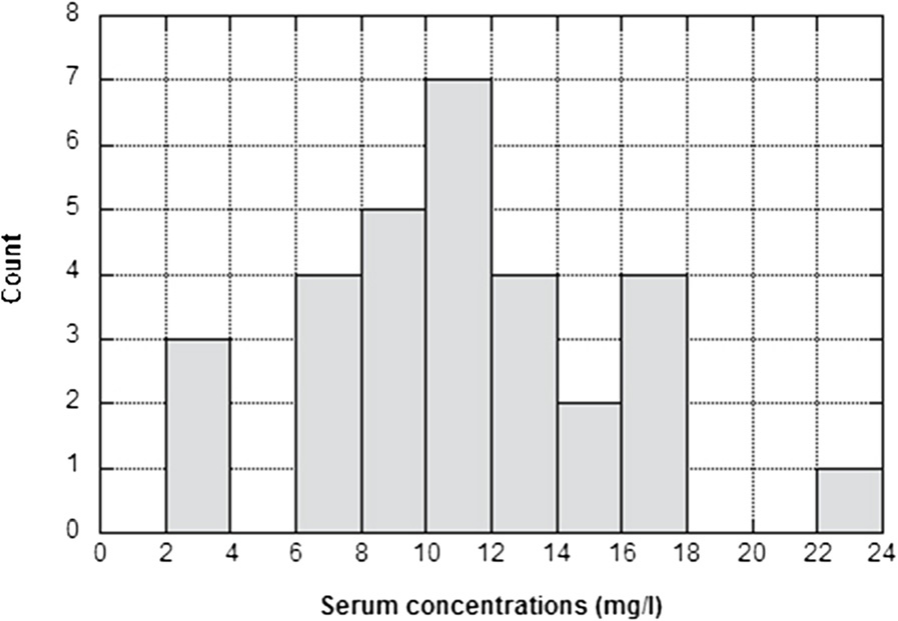
Distribution of the pre-dialysis serum cefepime concentrations (*n* = 30)

Overall, the mean dose of post-dialysis cefepime was 920 ± 270 mg (14.5 ± 5.1 mg/kg), but it was significantly lower before the 48-h interval (775 ± 210 mg or 12.7 ± 4.5 mg/kg) than before the 72-h interval (1125 ± 225 mg or 17.2 ± 4.9 mg/kg) (*p* < 0.05). The mean pre-dialysis cefepime concentrations were 10.7 ± 3.9 mg/l and 11.3 ± 5.6 mg/l at 48 and 72 h, respectively (*p* = NS). Fig. 1 shows the distribution of the pre-dialysis serum antibiotic concentrations and, with the exception of 3 values, indicates that all the residual pre-dialysis concentrations were higher than 6 mg/l. Table 1 shows that these levels always largely exceeded the EUCAST susceptibility breakpoints for all the targeted bacteria (>1 mg/l) with the exception of Pseudomonas aeruginosa for which the breakpoint is much higher (>8 mg/l). The MIC90 for the reference bacteria also was always reached except for P. aeruginosa.

**Table 1:** EUCAST susceptibility breakpoints (ref. 20) and MIC90 values (ref. 11) of the identified organisms

Bacteria	Breakpoint	MIC90
	(mg/l)	(mg/l)
Enterobacter cloacae	1	4
Serratia odorifera	1	0.5
Pseudomonas aeruginosa (*n* = 2)	8	16
Staphylococcus aureus	-	2
Klebsiella oxytoca	1	≤0.25
Acinetobacter sp.	-	-

We compared pre-dialysis cefepime serum levels of patients with residual diuresis to those of the anuric patients. While both groups received similar doses of cefepime (*p* = NS), pre-dialysis concentrations were significantly higher in the anuric patients when compared to those with preserved diuresis (15.6 ± 3.5 vs 9.25 ± 3.6 mg/l, *p* < 0.001). In the anuric patients, all the serum trough levels were >10 mg/l with only one value >18 mg/l (see Fig. 1).

In 17 HD sessions, cefepime serum concentrations were measured both before and after dialysis. Our data show that cefepime is quite effectively removed during dialysis with a mean 81.3 % reduction in serum levels from 10.5 ± 3.7 to 1.96 ± 1.2 mg/l (*p* < 0.001). In 77 % of the cases, post-HD concentrations remained above >1 mg/l.

The clinical course of all our patients was favorable and no significant side effects occurred. In two patients, antibiotic treatment was started during hospitalization and then continued after discharge. In all the other patients, treatment occurred entirely on an outpatient basis.

## Discussion

Chronic hemodialysis (HD) patients are at a high risk of developing infectious complications and infection is the second leading cause of death in this population after cardiovascular disease [1–5]. As a result of this high susceptibility to infection, dialysis patients often require hospitalization for prolonged treatment with IV antibiotics. In this regard, post-dialysis intravenous administration of antibiotics is quite interesting since it facilitates the management of even severe infections on an outpatient basis with a 100 % compliance [6–8], and thus improves the quality of life while reducing treatment costs. This explains why vancomycin has been largely used after dialysis for a long time in dialysis centers [9]. However, vancomycin has a narrow spectrum of action which limits its use to infections caused by Gram-positive microorganisms. Cefepime has a broader action spectrum [11, 12] and a pharmacokinetic profile that may permit intermittent post-dialysis administration three times each week [14, 18, 19] and is therefore of great interest from a clinical perspective.

Based on the pharmacokinetic studies with radiolabeled cefepime conducted by Barbhaiya et al., it had already been proposed in the early 1990s that cefepime could be prescribed once every 48 h in patients with severely impaired renal function [15]. In 1992, Cronqvist et al. studied the pharmacokinetics of 1 g of cefepime administered in volunteers with various degrees of renal impairment compared to healthy subjects and reported that elimination of cefepime is mainly renal and correlates closely and linearly to GFR with negligible tubular secretion [14]. In five HD patients, the maximum serum concentration after antibiotic infusion was slightly higher (130 ± 23 mg/l) than in normal subjects. However, the drug clearance was 15 times lower, the elimination half-life was 12 times as long (up to 22 h) and the area under the concentration-time curve (AUC) was 18 times higher [14]. In 2000, Schmaldienst et al. reported on the pharmacokinetics of cefepime in 6 anuric HD patients who were treated with high-flux dialyzers and received a fixed dose of 2 g after each HD. In these patients, the mean peak serum concentration was 166 ± 49 mg/l and the mean trough levels were 23 ± 7 mg/l [18]. Based on their results, these authors proposed the administration of a fixed dose of 2 g three times each week in anuric HD patients. Five years later, this same group reported on its clinical experiences in treating 11 anuric HD patients according to that protocol and reported a success rate of 82 % [19].

Although this Austrian group did not report significant side effects in their patients, the relatively high cefepime trough plasma concentrations which they measured were initially of concern to us because severe neurological side effects have been described with high levels of several beta-lactam antibiotics including cefepime [20–22]. Recently, Lamoth et al. reported that the probability of cefepime-associated neurological toxicity (i.e. altered mental status, confusion, or myoclonia) increases steadily with trough plasma levels exceeding 22 mg/l [21]. Another concern regarding the regimen proposed by the Austrian group is the fixed antibiotic dose which is administered three-times-weekly. Our previous experience with post-dialysis ceftriaxone administration [10] has shown us that generally higher doses are required before a 3-day weekend interval in order to achieve sufficient trough plasma concentrations at the end of this period. Finally, these authors limit their use of post-dialysis cefepime to anuric patients which - of course - is not the condition of a large number of HD patients, particularly among those starting on a HD program [25].

Consistent with the concerns discussed above, we realized that an approach based on the therapeutic drug monitoring (TDM) concept should facilitate the prescription of a post-dialysis dose that is high enough to ensure adequate trough plasma concentrations during the entire one-week period while avoiding potentially toxic cefepime levels. As shown in the results section, with the doses of cefepime we prescribed the trough plasma concentrations were well above the EUCAST breakpoints for susceptible organisms in most individual patients and above the MIC90 of a relevant clinical collection of bacteria with the exception of P. aeruginosa. Using our approach, only one of our anuric patients (on one occasion) had a trough level higher than 18 mg/l. It is worth noting that the cefepime doses which we used in our patients were on average ≈ 50 % lower than the fixed dose of 2 g after each dialysis proposed by the aforementioned Austrian group [18, 19].

Mean cefepime concentrations were significantly lower in our non-anuric patients. These lower concentrations observed in patients still having residual diuresis suggest that a certain amount of cefepime is still excreted in urine and correlates with the fact that most of its elimination is related to the glomerular filtration rate [14]. Therefore, in patients with residual diuresis the prescription of higher doses of cefepime should be considered in order to achieve plasma trough levels that are high enough. This point is clearly crucial in patients who are suffering from an infection caused by less susceptible bacteria such as P. aeruginosa.

Finally, it should be noted that several previous studies have already reported that cefepime is effectively cleared during dialysis with a reduction rate ranging from 40 to 68 % during 3-h sessions [14, 18, 22]. Our data shows that a 4-h session with high-flux dialyzers reduces the drug by about 80 %. This point may be of interest to patients who are suffering from side effects related to cefepime accumulation (which occurs most often in patients with renal dysfunction) and who can rapidly improve after a single dialysis session [21, 22].

This study has some limitations. First, it is a retrospective cohort study conducted at a single center. Second, it concerns a small number of patients. However it should be noted that all the patients were accurately monitored and that, on average, 4 to 5 determinations of the cefepime serum levels were performed in each patient. Also, a strength of the present study is that it is the first one that reports the data concerning the clinical use of post-dialysis cefepime based a therapeutic drug monitoring (TDM) approach. It should be noted, however, that a potential limitation of this approach is the fact that cefepime serum level measurement is not widely available at present time.

## Conclusions

In conclusion, outpatient treatment with cefepime administered after dialysis three-times-weekly proved to be effective and well-tolerated in our patients while reducing hospitalization and improving their quality of life. According to the presented data, in patients having infections with highly susceptible pathogens a fixed dose of cefepime of 1 g before every 48-h interval and of 1.5 g before every 72-h interval can be recommended, without need of routine monitoring of the cefepime blood levels. However in patients with suspected or proven infection with less susceptible pathogens as P. aeruginosa and particularly in those patients exhibiting significant residual renal function higher initial doses should be prescribed, i.e. 1.5 g before a 48-h interval and 2 g before a 72-h interval. In this latter group of patients the quantification of the drug’s levels is mandatory in order to adapt the subsequent doses to the patient’s trough serum levels.

## Abbreviations

CKD: chronic kidney disease
EUCAST: European Committee on Antimicrobial Susceptibility Testing
HD: hemodialysis
IV: intravenous
MIC90: Minimum Inhibitory Concentration for 90 % of organisms
TDM: therapeutic drug monitoring
